# Evaluating patient data quality in South Africa’s National Health Laboratory Service Data Warehouse, 2017-2020: implications for monitoring child health programmes

**DOI:** 10.1186/s12889-022-13508-y

**Published:** 2022-06-29

**Authors:** Lebohang Radebe, Ahmad Haeri Mazanderani, Gayle G. Sherman

**Affiliations:** 1 Centre for HIV and STIs, National Institute for Communicable Diseases, National Health Laboratory Service, 1 Modderfontein Road, Sandringham, Johannesburg, 2131 South Africa; 2 Paediatric HIV Diagnostics Division, Wits Health Consortium, Johannesburg, South Africa; 3 Department of Paediatrics and Child Health, Faculty of Health Sciences, University of the Witwatersrand, Johannesburg, South Africa

**Keywords:** Unique patient identifier, Data quality, Monitoring and evaluation, Public health programmes, Early infant HIV diagnosis

## Abstract

**Background:**

South Africa’s National Health Laboratory Service (NHLS), the only clinical laboratory service in the country’s public health sector, is an important resource for monitoring public health programmes.

**Objectives:**

We describe NHLS data quality, particularly patient demographics among infants, and the effect this has on linking multiple test results to a single patient.

**Methods:**

Retrospective descriptive analysis of NHLS data from 1^st^ January 2017—1^st^ September 2020 was performed. A validated probabilistic record-linking algorithm linked multiple results to individual patients in lieu of a unique patient identifier. Paediatric HIV PCR data was used to illustrate the effect on monitoring and evaluating a public health programme. Descriptive statistics including medians, proportions and inter quartile ranges are reported, with Chi-square univariate tests for independence used to determine association between variables.

**Results:**

During the period analysed, 485 300 007 tests, 98 217 642 encounters and 35 771 846 patients met criteria for analysis. Overall, 15.80% (*n* = 15 515 380) of all encounters had a registered national identity (ID) number, 2.11% (*n* = 2 069 785) were registered without a given name, 63.15% (*n* = 62 020 107) were registered to women and 32.89% (*n* = 32 304 329) of all folder numbers were listed as either the patient’s date of birth or unknown. For infants tested at < 7 days of age (*n* = 2 565 329), 0.099% (*n* = 2 534) had an associated ID number and 48.87% (*n* = 1 253 620) were registered without a given name. Encounters with a given name were linked to a subsequent encounter 40.78% (*n* = 14 180 409 of 34 775 617) of the time, significantly more often than the 21.85% (*n* = 217 660 of 996 229) of encounters registered with a baby-derivative name (*p*-value < 0.001).

**Conclusion:**

Unavailability and poor capturing of patient demographics, especially among infants and children, affects the ability to accurately monitor routine health programmes. A unique national patient identifier, other than the national ID number, is urgently required and must be available at birth if South Africa is to accurately monitor programmes such as the Prevention of Mother-to-Child Transmission of HIV.

**Supplementary Information:**

The online version contains supplementary material available at 10.1186/s12889-022-13508-y.

## Background

The National Health Laboratory Service (NHLS) is the largest and sole diagnostic pathology service provider to the South African public health sector, serving over 80% of the population [1]. As a unique source of public health information, NHLS data has been used to monitor and evaluate numerous diseases and public health programmes including tuberculosis [2], Hepatitis A [3], syphilis [4] and HIV [5–8]. Furthermore, NHLS data has been validated against costly national surveys monitoring the Prevention of Mother-to-Child Transmission (PMTCT) of HIV programme and found to yield accurate results, thereby demonstrating its cost-effective utility for routine public health surveillance [9].

Despite being a rich source of information, important limitations exist with the use of routine laboratory data. In particular, the absence of a national unique patient identifier (UPI) hampers the ability to analyse patient-level data. To overcome this challenge, a probabilistic record-linking algorithm is employed by the NHLS Corporate Data Warehouse (CDW) to de-duplicate test-level data. This algorithm has been found to have a sensitivity of 73% and a positive predictive value of 83% in matching adult HIV test data [10], with good accuracy and completeness of the test-level data reported [11]. However, the performance of the NHLS CDW algorithm has been found to vary across diagnostic programmes, with considerable under-linking of infant HIV test results reported [12].

In this analysis, we evaluate patient identifying details within the NHLS data warehouse and describe the extent of specimens submitted for routine laboratory testing that are registered without a given name (i.e. have a baby-derivative name), sex or folder number. Furthermore, the implications this has on linking subsequent test results are evaluated, with the HIV early infant diagnosis (EID) programme used as an example to describe the impact this has on monitoring routine child health programmes.

## Methods

### Design

This is a retrospective descriptive analysis of routinely collected laboratory data from the NHLS. All test results, registered between 1^st^ January 2017 and 1^st^ September 2020 were extracted for analysis. In terms of identifiers, we extracted test episode numbers and the UPI generated by the CDW algorithm. We used the episode number, which is generated whenever a sample is registered on the NHLS laboratory information system, to estimate the number of patient encounters with the NHLS during which a number of different tests can be performed. In terms of test information, we extracted the date the test was registered, the name of the test and the facility location where the patient was seen, including the names of the facility, sub-district, district and province. Finally, we extracted demographics of the patient, including the patient’s first and last name, their date of birth, sex and folder number (an alphanumeric identifier used by facilities to identify a patient). In addition to the extracted variables, we derived four categorical variables – age (< 7 days, 7 days – < 3 months, 3 months – < 2 years, ≥ 2 years), facility type (clinic, hospital, or unknown), folder number type (valid, date of birth used as folder number, or unknown) and name type (either a given name for both first and last name or derivative of ‘baby to’ for either first or last name—referred to hereon as baby-derivative name). We describe the reason for each variable and its derivation in Supplementary Table 1.

### Linking algorithm

The NHLS CDW assigns the same UPI to all episode numbers likely belonging to the same patient, utilising an unpublished, in-house developed probabilistic record-linking algorithm. An individual is distinguished from another based on first name, last name, and date of birth as determined by exact linking. Where available, two additional attributes are utilised for exact linking—namely, the South African national identification (ID) number and patient folder number [5]. The latter is restricted to facilities in the Western Cape and academic hospitals where the folder number is a reliable unique identifier. For episodes not linked by the exact linking stored procedure, fuzzy-logic linking is applied, utilising first name, last name, and date of birth. The purpose of this additional step is to provide more accurate linking by accommodating different spelling of names and transcription/typographical errors. However, the overall validity of this approach is unknown.

Given that the name features so heavily in the linking algorithm, one way of examining who is being linked is by looking at the effect of linking by name type. The Western Cape Province is the only province that assigns each person a provincial folder number that is used to link patient records across the whole province regardless of which healthcare facility they visit [13]. Thus, it is incorporated into the CDW algorithm for tests emanating from that province and becomes an important variable to analyse the quality of linking. In the other provinces, facility numbers are specific to the healthcare facility attended.

### Setting, participants and main outcome measures

We extracted all test level data captured in the NHLS between 1^st^ January 2017 and 1^st^ September 2020. This included all tests ordered from primary, secondary and tertiary public health facilities in all nine provinces. We excluded tests if they were not associated with a CDW UPI (e.g. tests conducted as part of a study), were non-human, environmental or animal samples, and if they had invalid ages, defined as < 0 years old or ≥ 120 years old. Birth tests were defined as any test conducted on a child < 7 days old.

To better understand the impact demographic details have for monitoring public health programmes during infancy, additional analysis was performed on HIV polymerase chain reaction (PCR) results. Since June 2015, national guidelines have recommended that every HIV-exposed child (approximately 30% of all live births) [14] receive an HIV PCR test at birth, at 10 weeks of age and after cessation of breast-feeding. If a patient tests positive, then a confirmatory HIV PCR test and immediate initiation of antiretroviral therapy is recommended.

We report percentages as proportions of total tests/patients per sex, age, province, facility type, folder number type and test type. Where medians are reported, the first and third quartile are reported in brackets. All statistical analysis, including counts and percentages, were performed using IBM Netezza and maps were generated using QGIS 3.12.1. Pearson’s Chi-square univariate tests for independence were performed using R version 3.6.3 on a 64-bit Windows.

#### Ethical considerations

The National Institute for Communicable Diseases has been granted ethics approval to conduct communicable disease surveillance and analysis of routine laboratory data by the Human Research Ethics Committee of the University of the Witwatersrand (M160667; M210752). All study methods were performed in accordance with the relevant guidelines and regulations, with requirements for informed consent waived by the Human Research Ethics Committee of the University of the Witwatersrand. Only the authors accessed patient identifiers, and all analysis with identifiable data was performed on a secure password-protected server located on the NHLS campus. All downloaded data were in summary and therefore unidentifiable, and were password protected.

## Results

Between 1^st^ January 2017 and 1^st^ September 2020, the NHLS registered 504 040 400 tests from 102 487 788 encounters associated with 39 637 704 patients of which 485 300 007 (96.48%) tests, 98 217 642 (96.17%) encounters and 35 771 846 (91.03%) patients met criteria for analysis. Of all the encounters, 15.80% (15 515 380 of 98 217 642) had a registered ID number, dropping to 0.099% (2 534 of 2 565 329) of tests conducted at < 7 days of age.

### Overall trends

#### Ages

Table 1 describes the testing pattern for all tests by age. In terms of all tests in the NHLS during this period, testing in under two year olds accounted for 6.77% (*n* = 32 874 962), 6.96% (*n* = 6 834 774) and 8.75% (*n* = 3 643 371) of all tests, encounters and patients, respectively. Restricting to just < 2 years old, birth testing (< 7 days) accounted for 32.85% (*n* = 10 794 234), 37.53% (*n* = 2 565 329) and 42.19% (*n* = 1 537 277) of all tests, encounters and patients, respectively.

**Table 1:** Number of tests, encounters and patients per age group

Ages	Tests, Number (Column %)	Encounters, Number (Column %)	Patients, Number (Column %)
< 7 days	10 794 234 (2.22%)	2 565 329 (2.61%)	1 537 277 (4.30%)
7 days – < 3 months	10 881 816 (2.24%)	2 280 909 (2.32%)	1 186 128 (3.32%)
3 months – < 2 years	11 198 912 (2.31%)	1 988 536 (2.02%)	919 966 (2.57%)
≥ 2 years	452 425 045 (93.23%)	91 382 868 (93.04%)	32 642 382 (91.25%)
**Total**	**485 300 007**	**98 217 642**	**35 771 846**^a^

^a^ Not sum of four groups since one person can have encounters in more than one age group

The top five tests for each age group is described in Table 2. HIV PCR testing was the most common test performed among < 7 day olds (*n* = 939 559, 8.70%) whereas the most common test among the ≥ 2 year olds was creatinine (*n* = 40 874 061, 9.03%) followed by HIV viral load (*n* = 20 234 750, 4.47%).

**Table 2:** Top five tests per age group, 2017- 2020

Rank	Ages (% of tests in age group)			
	< 7 days	7 days – < 3 months	3 months – < 2 years	≥ 2 years
1	HIV-1 Qualitative PCR (8.70%)	HIV-1 Qualitative PCR (7.31%)	Potassium (5.22%)	Creatinine (9.03%)
2	Total Bilirubin (5.75%)	Sodium (4.53%)	Sodium (5.19%)	HIV Viral Load (4.47%)
3	Conjugated Bilirubin (5.06%)	Potassium (4.48%)	Urea (5.16%)	Potassium (4.18%)
4	C-reactive Protein (4.43%)	Urea (4.45%)	Creatinine (5.14%)	Urea (4.11%)
5	Full Blood Count (4.25%)	Creatinine (4.34%)	Full Blood Count (4.49%)	Sodium (4.00%)

#### Sex, facility type and folder number type

**Fig. 1:**
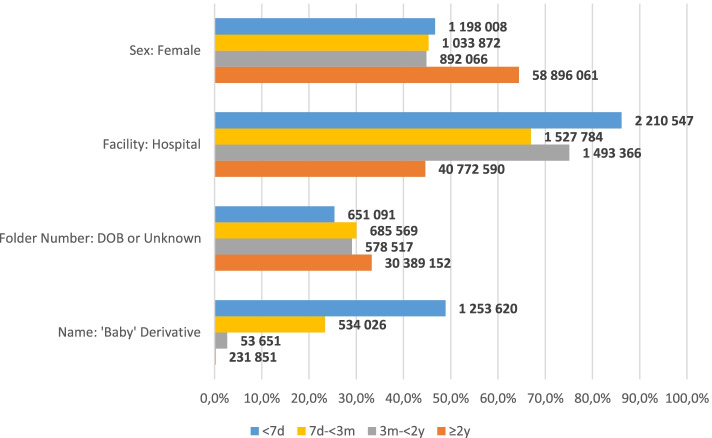
Trends in sex, facility type, folder number type and name by age. d, days; m, months; y, years

Figure 1 details the trends in sex, facility type, folder number type and name type by age. Women were tested more frequently than men, accounting for 63.15% (*n* = 62 020 107) of all encounters. This trend was not mirrored in those aged < 2 years, where 45.71% (*n* = 3 124 046) of all encounters were registered as female. As age increased, the percentage of encounters registered with unknown sex decreased from 5.25% (*n* = 134 769) among those aged < 7 days, to 4.33% (*n* = 98 694) among 7 days—< 3 months, to 3.62% (*n* = 72 050) 3 months—< 2 years, to 1.38% (*n* = 1 256 893) among the ≥ 2 year olds.

Overall, testing was split almost evenly between hospitals and clinics, with 46.84% (*n* = 46 004 287) of encounters registered from hospitals, and 45.49% (44 675 032) registered from clinics, with 7.69% (7 538 323) unable to be assigned. However, a much larger proportion of patients < 2 years were tested in hospital (76.55%). Overall, 32.89% (*n* = 32 304 329) of folder numbers were listed as either the patient’s date of birth or unknown. This trend was consistent across all age groups.

#### Geospatial distribution & name type

**Fig. 2:**
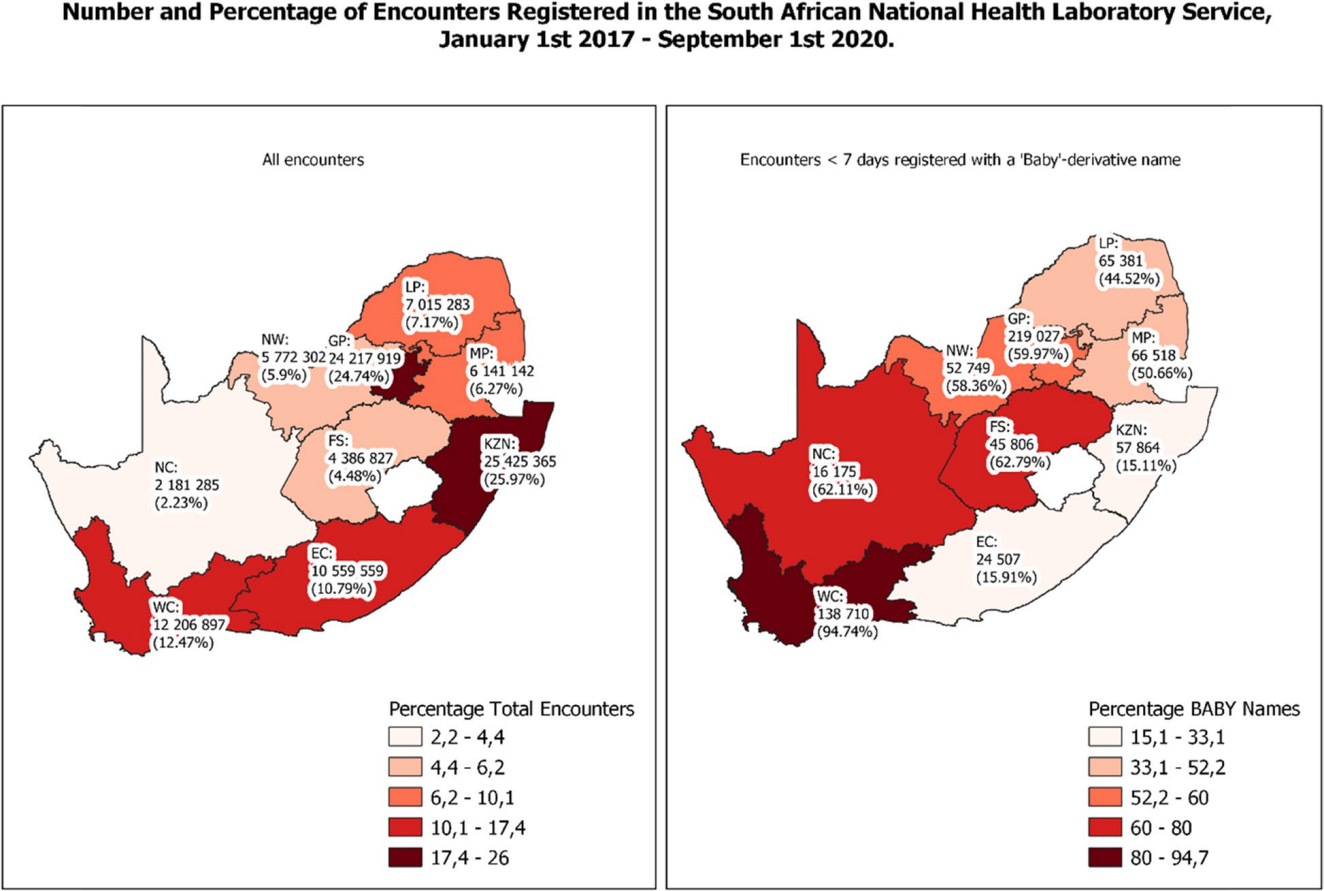
Geospatial distribution of NHLS encounters

Testing volumes followed the same pattern as population density across the country (Fig. 2) [15]. Regarding patient names, ‘Baby’ was the third most common name among encounters of all ages, occurring 400 326 times (0.41%). While some patients are genuinely named ‘Baby’, when restricting to just ≥ 2 year olds, the popularity of this name decreased to 1 137^th^ place and occurred just 12 842 times (0.01%).

Overall, 2 069 785 encounters (2.11%) were registered with a baby-derivative name. Table 3 describes the top five first names amongst all encounters for all four age groups as well as the percentage with a baby-derivative name, which decreased as patient age increased. The national percentage of baby-derivative encounters was highest for those aged < 7 days at 48.87% (*n* = 1 253 620). However, the percentage by province varied considerably from a maximum of 94.74% (*n* = 138 710) in the Western Cape to a minimum of 15.11% (*n* = 57 864) in KwaZulu-Natal (Fig. 2).

**Table 3:** Top 5 first names per age group, 2017 – 2019

	Ages (% of name in age group)			
Rank	< 7 days (48.87%^a^)	7 days – < 3 months (23.41%^a^)	3 months – < 2 years (2.70%^a^)	≥ 2 years (0.25%^a^)
1	**Baby** (9.85%)	**Baby** (5.52%)	Enzokuhle (0.71%)	Maria (0.72%)
2	**Baby Boy** (0.68%)	**Baby Boy** (0.57%)	Melokuhle (0.65%)	Elizabeth (0.53%)
3	**Baby Girl** (0.59%)	Enzokuhle (0.53%)	Lubanzi (0.47%)	Zanele (0.39%)
4	**BT** (0.40%)	**Baby Girl** (0.47%)	**Baby** (0.45%)	Johannes (0.35%)
5	Zanele (0.37%)	Melokuhle (0.45%)	Lethabo (0.41%)	Lindiwe (0.34%)

^a^ Total percentage of baby-derivative name in age group. Baby-derivative names in bold

#### Follow-up testing

Overall, encounters registered with a baby-derivative name were significantly less likely to be linked to another encounter compared to encounters registered with a given name; 21.85% (217 660 of 996 229) versus 40.78% (14 180 409 of 34 775 617) (*p*-value < 0.001). This finding was consistent across all age groups. In terms of age at first encounter, 29.09% (*n* = 441 139 of 1 516 709) of all initial encounters < 7 days of age were linked with a subsequent encounter versus 41.49% (*n* = 13 522 534 of 32 588 823) for patients whose first encounter occurred at ≥ 2 years old (*p*-value < 0.001).

#### Name and facility type

Among all linked test results, the first and last name listed on both encounters were exactly the same (i.e. not a single character difference for both first name and surname) for 83.15% (*n* = 180 987) of baby-derivative named encounters and 83.65% (*n* = 11 862 312) for given named encounters. For those initially registered with a baby-derivative name, only 8 414 (3.87%) were subsequently linked to an encounter with a given name.

Regardless of the facility type at which the initial encounter took place, the percentage of linked follow-up tests was roughly the same at 40.73% (*n* = 5 653 400 of 13 881 448) for hospitals versus 40.58% (*n* = 7 531 940 of 18 562 506) for clinics. However, a significantly greater proportion of patients with a baby-derivative name were tested in a hospital (*n* = 746 811, 74.96%) versus those with a given name (*n* = 13 134 637, 37.77%) (*p*-value < 0.001).

#### Folder number type: Western Cape versus all other provinces

**Fig. 3:**
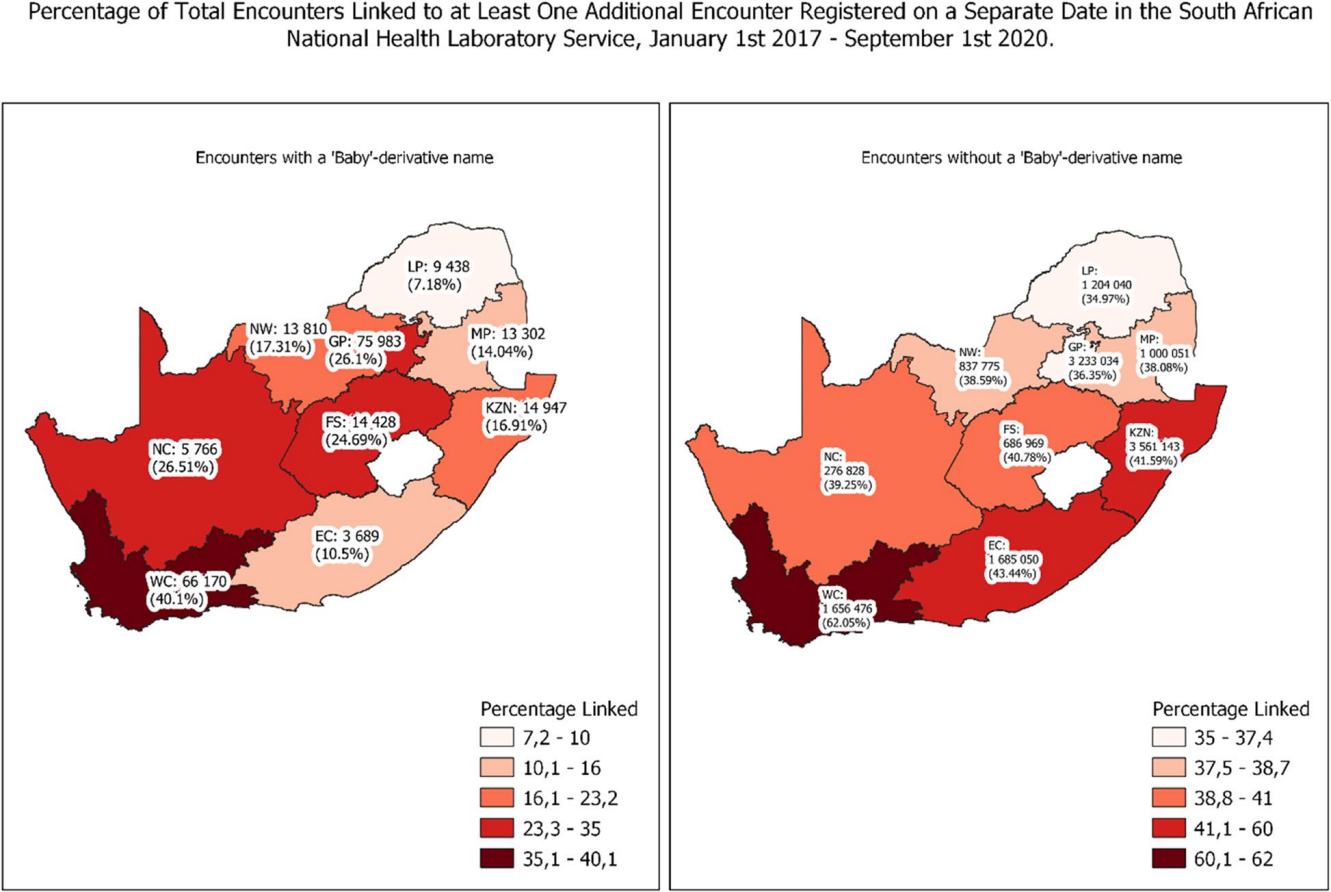
Geographic Distribution of Linked Tests < 7 days with and without a baby-derivative name

Whereas all provinces have a greater percentage of linked encounters among tests registered with given names compared with baby-derivative names (Fig. 3), the Western Cape, which uses a provincial folder number per patient across all facilities, had the greatest percentage of linked encounters for both given (*n* = 1 656 476, 62.05%) and baby-derivative names (*n* = 66 170, 40.13%). In the rest of the country where no higher-level folder number exists, this dropped to 39.00% (*n* = 12 522 856 of 32 106 176) for initial given name encounters and 18.22% (*n* = 151 473 of 831 284) for initial baby-derivative name encounters. In addition, the Western Cape had the largest percentage of birth tests with a baby-derivative name linked with at least one subsequent encounter on a separate date (*n* = 59 800, 43.11%). This is despite the fact that 94.74% (*n* = 138 710) of all birth tests in the province were registered with a baby-derivative name (Fig. 2).

### HIV Qualitative PCR testing

Between January 1^st^ 2017 and September 1^st^ 2020, 2 266 606 HIV PCR tests were performed on 1 948 501 patients. Overall, 430 831 (22.11%) were registered with a baby-derivative name, of which 387 095 (89.8%) were birth tests. Testing volumes, both overall and those associated with baby-derivative names, are presented by age at testing in Table 4.

**Table 4:** HIV-1 PCR qualitative tests, 1 January 2017 – 1 September 2020

Ages	Total		Name Type: baby-derivative	
	**Tests**, n (column %)	**Patients**, n (column %)	**Tests**, n (row %)	**Patients**, n (row %)
< 7 days	939 559 (41.45%)	917 137 (47.07%)	399 262 (42.49%)	387 095 (42.21%)
7 days – < 3 months	795 811 (35.11%)	759 062 (39.00%)	46 422 (5.83%)	44 755 (5.90%)
3 months – < 2 years	480 231 (21.19%)	435 683 (22.36%)	11 209 (2.33%)	10 368 (2.38%)
≥ 2 years	51 005 (2.25%)	49 842 (2.56%)	5 194 (10.18%)	5 144 (10.32%)
**Total**	**2 266 606**	**1 948 501**^a^	**462 087 (20.39%)**	**430 831**^a^ **(22.11%)**

^a^ Not sum of four groups since one person can have two HIV PCR tests in different age groups in during the period analysed

#### Follow-up testing and positives

Among all patients with an HIV PCR test, 398 875 (20.47%) had a linked subsequent test, of which 51 724 (12.97%) had an initial baby-derivative name and 347 151 (87.03%) had an initial given name. The median time to follow-up testing was longer for patients with a given name, of 70 days (IQR: 27–83), compared to those with a baby-derivative name, of 2 days (IQR: 1–64).

**Fig. 4:**
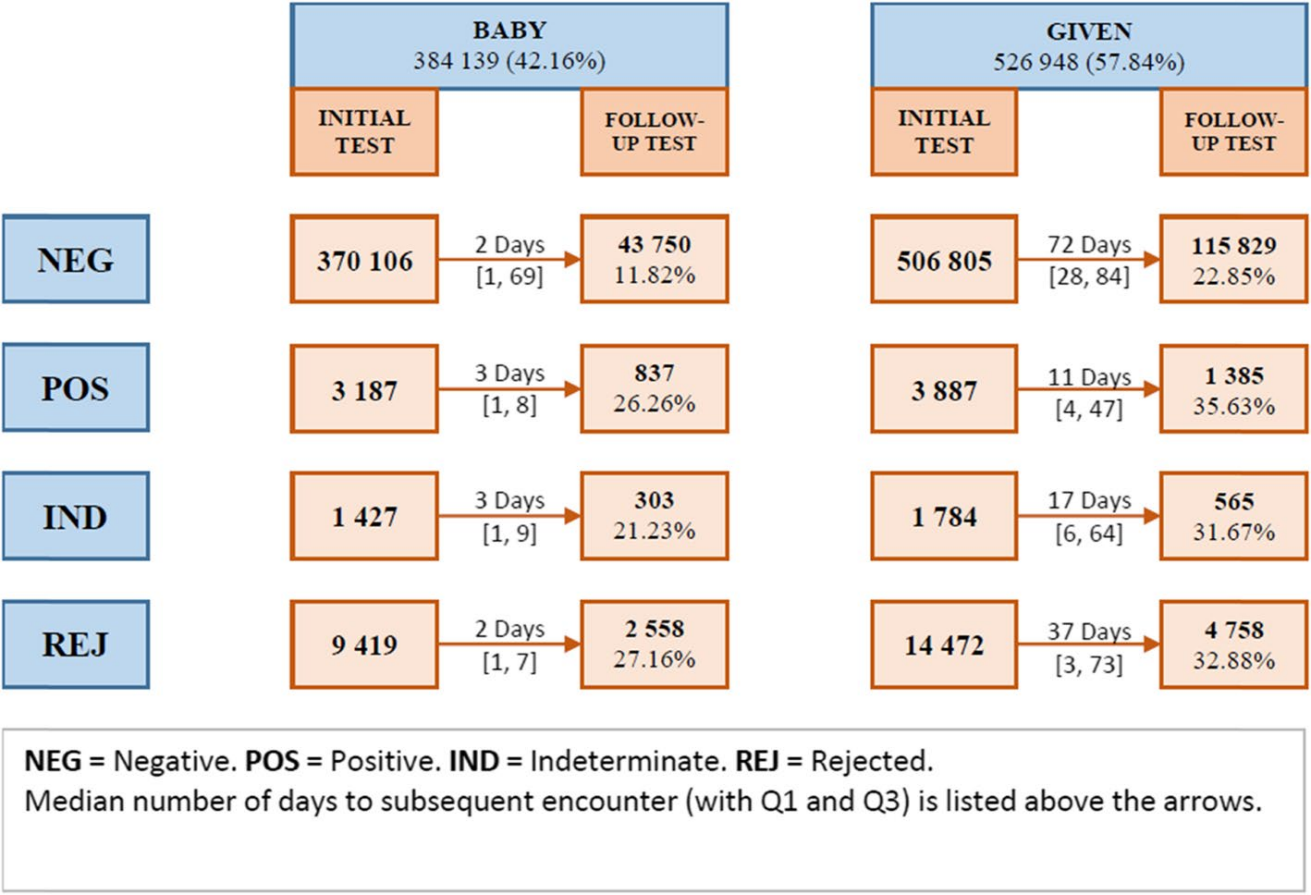
Number and percentage of HIV PCR follow-up tests for initial encounters < 7 days by name type and test result

Overall, there were 32 528 (1.70%) patients who had at least one positive test, of which 7 206 (22.15%) received their first positive at < 7 days, 9 477 (29.13%) at 7 days – < 3 months, 12 773 (39.27%) at 3 months—< 2 years and 3 072 (9.44%) at ≥ 2 years of age. Of the total, 4 106 (12.62%) had an initial baby-derivative named encounter, while the remaining 28 422 (87.38%) had a given name at the first encounter. The overall difference in linking a positive result with a subsequent confirmatory result amongst those with a baby-derivative name versus those with a given name was significant at 27.93% (*n* = 1 147) for baby-derivative names versus 62.14% (n = 17 661) for given names (*p*-value < 0.001). This suggests that the number of infected infants first diagnosed between 7 days—< 3 months is likely overestimated because in this age group, more patients have given names that would not have linked to their previous baby-derivative names from the < 7 day age group. Figure 4 provides the follow-up testing numbers and proportions by initial HIV PCR result and registered name for birth tests (< 7 days). The 759 062 (82%) infants tested at 7 days—< 3 months should be approximately the same 917 137 HIV-exposed neonates tested at birth (Table 4), considering guidelines recommend testing at birth and then at 10-weeks of age (if the birth test is negative) and birth testing coverage is > 94% of all HIV-exposed infants [8]. Yet, an initial negative HIV PCR test was only linked to a later test in the same child in 22.85% of cases (Fig. 4) suggesting substantial under-linking by the algorithm. The higher proportion of linkages in a shorter duration of time for positive, indeterminate and rejected HIV PCR results are likely related to clinical guidelines that recommend that a second HIV PCR test be performed as soon as possible thereafter. Additionally, tests performed in rapid succession are likely to have the same names (given or baby derivatives) facilitating record linking and allowing for proportionately more HIV PCR positive than negative children to be linked. Linkage of between 26%—36% of first positive PCR tests performed at < 7 days of age may reflect poor algorithm performance and/or poor implementation of confirmatory PCR testing and linkage of HIV-infected neonates to care.

## Discussion

This is the first description of data quality pertaining to patient details within South Africa’s NHLS data warehouse, and how it relates to the ability to de-duplicate test-level data, link patient results and longitudinally monitor child health programmes. Our findings suggest that patient information is better recorded among older patients compared with infants and young children – and is especially poor among new born infants for whom half of all tests are registered with a baby-derivative name. Where given names are captured their frequency is corroborated by the top baby names as reported by Statistics South Africa [16], further highlighting the nationally representative nature of data housed within the NHLS data warehouse.

Importantly, consistent capturing of accurate patient information improves the ability to link patient records, with tests registered with a baby-derivative name half as likely to be linked to a subsequent result when compared with tests registered with a given name. An exception to this is in the Western Cape Province, where linking is higher compared to the national average regardless of the name listed on the initial encounter. This is due to utilisation of a provincial folder number, whereby an individual maintains the same folder number at every encounter with the healthcare system. This enables very strong linking to occur regardless of patient demographic information.

In the absence of a national UPI available at birth and accessible at subsequent healthcare visits, the above findings highlight an important limitation with using routine laboratory data for monitoring child health programmes. These programmes include evaluating PMTCT for HIV and syphilis, as well as determining the burden of congenital rubella and hepatitis B infections – data which will prove increasingly relevant as South Africa prepares to include rubella and hepatitis B birth-dose vaccinations into its Expanded Programme on Immunisation [17, 18]. As we have demonstrated with HIV PCR data, it is imperative that a UPI be available at time of birth testing to ensure consistent linkage of subsequent test results. As this remains wanting at a national level, the number of infants and children diagnosed with HIV in South Africa, and the extent of loss to follow-up among these patients, remains unknown. This has serious implications not only for disease surveillance but also individual patient care.

The National Department of Health’s Health Patient Registration System (HPRS), which has been developed to provide a patient registry using the South African ID number, is not fit for purpose when it comes to accommodating early infant programmes. South African ID numbers are not universally available at time of birth testing and delay in naming of infants may further delay issuing of a birth certificate, and hence ID number [19]. Furthermore, national ID cards are only issued at age 16 years and older and mothers do not routinely carry their infant’s birth certificates to healthcare visits making the ID number inaccessible for use in children even after early infancy. Whereas the Road to Health Booklets (RTHB), the patient-held immunisation and growth record used up until 5 years of age, has a dedicated field for capturing ID numbers, the completeness of RTHBs has been found to be suboptimal [20]. Consistent use of provincial folder numbers, as demonstrated by the Western Cape Department of Health, represents an alternative. However, this would require an overhaul of current practice across all public healthcare facilities considering that a third of all tests registered were found to use the patient’s date of birth or had an unknown folder number. Other solutions include leveraging the RTHB to provide a stop-gap whereby RTHBs are issued with unique barcoded stickers that can be incorporated in the laboratory information system, as demonstrated by a pilot project from Tshwane District [12]. These RTHB identifiers can then be linked with the South African ID number when issued to enhance the utilization of the HPRS for monitoring child health programmes. A UPI available at birth would also facilitate linkage of mother-infant pairs for longitudinal cohort monitoring of the PMTCT programme.

A few important limitations need to be considered regarding this analysis. Test results were linked using a probabilistic record-linking algorithm relying heavily on name and surname, and hence limited by the accuracy of the algorithm. Although the CDW algorithm has demonstrated good accuracy within the adult HIV programme [11], the ability to link baby-derivative names is clearly negatively influenced by inconsistent capturing of patient demographics. This in turn causes other variables to be associated with linkage of test results, such as time to follow-up testing, on account of more consistent capturing of name and folder number during a single patient admission. Whereas alternative and better performing algorithms have been described [21], these are still critically limited by their inability to link records of infants with different names captured at different visits and no other patient identifier consistently used. Furthermore, as there is no national gold standard data-set with which to evaluate true follow-up testing patterns within the NHLS, it is not possible to determine the true extent to which record-linking algorithms under-estimate patient follow-up. However, as the EID data reported in this analysis suggests that over 80% of infants with a birth HIV PCR test had repeat testing between 7 days – < 3 months of age, it is reasonable to conclude that less than a third of birth PCR results (possibly as few as a quarter) were linked to subsequent test records, even among those with a given name at birth, using the CDW algorithm. Finally, although it is reasonable to extrapolate poor record-linking within the EID programme to birth tests performed for other conditions, such as congenital syphilis and rubella, the performance of record-linking algorithms among these test types is unknown.

## Conclusions

In summary, routine laboratory data represents an invaluable opportunity for monitoring health programmes cost-effectively and in near real-time. However, poor quality of patient demographic data negatively affects the ability to link patient test results within the NHLS data warehouse thereby limiting its utility for surveillance purposes. This is particularly problematic for infants and young children, where patient information is missing more often than adult populations. South Africa’s implementation of a unique patient identifier in the Health Patient Registration System needs to accommodate infant testing, taking into account the limited availability of national ID numbers and given names available at birth.

## Supplementary Information

**Additional file 1.**

## Data Availability

The datasets analysed during the current analysis are not publicly available due to them containing information that could compromise participant privacy including patient first and last name, gender, location and test conducted. Please contact the South African National Health Laboratory Service for data requests (https://aarms.nhls.ac.za/NHLS_AARMS/Public/Default.aspx).

## Abbreviations

NHLS: National Health Laboratory Service
PMTCT: Prevention of Mother-to-Child Transmission of HIV programme
CDW: Corporate Data Warehouse
EID: HIV Early Infant Diagnosis programme
UPI: Unique Patient Identifier
RTHB: Road to Health Booklet
