# Supplementary material for: Evaluating patient data quality in South Africa’s National Health Laboratory Service Data Warehouse, 2017-2020: implications for monitoring child health programmes
Source: BMC Public Health. 2022 Jun 29;22:1266. doi: 10.1186/s12889-022-13508-y (PMC9241268; doi:10.1186/s12889-022-13508-y)
Supplement: Supplementary file 1 — Additional file 1. [file 12889_2022_13508_MOESM1_ESM.docx]

| **Variable Name** | **Reason** | **Levels** |
| --- | --- | --- |
| Age | Used to analyse paediatric testing pattern. Based on recommended ages of routine HIV testing in the National Early Infant Diagnosis Program. | - *< 7 days* - *7 days – < 3 months* - *3 months – < 2 years* - *≥ 2 years* |
| Facility Type | Used to indicate whether a patient received care at a primary or secondary/tertiary institution. Based on facility name where sample was taken. | - *Clinic*: ‘CLINIC’, ‘CHC’, ‘CDC’ - *Hospital*: ‘HOSPITAL’ - *Other*: neither *Clinic* nor *Hospital* |
| Folder Number Type | Used to identify the type of file number received by each patient at the facility where they are tested. Based on iterative manual identification and selection of date of birth* (DOB) and Unknown patterns in the folder numbers. | - *DOB*:* YYYYMMDD, DDMMYY, YY0101, YY/MM/DD, DD/MM/YY, MM/YY, YY/MM - *Unknown*: ‘UNKNOWN’, ‘?’, ‘N/A’, ‘NO FILE NO’, ‘NO F/N’, ‘NO FILE’, ‘NO FIOLE’, ‘NOT STATED’, ‘12345678’, ‘BLOOD GAS’, ‘GLOBAL FUND’, ‘AURUM INSTI’, ‘NP’, ‘STAFF’, ‘ANC’, ‘PHC’, ‘OFFLINE’, ‘AURUM’, TMRH12390’, ‘NOT PROVIDED’, ‘LPSEY’, ‘PRH’, ‘THCA MOBILE’, ‘N/S’ - *Likely Valid*: neither *DOB* nor *Unknown* |
| Name Type | Used to identify whether the first and last name of a patient are given (GIVEN), or whether at least one is unknown (BABY). Based on iterative manual identification and selection of BABY patterns in the first and last name. | - *Baby-derivative name***: First name - '%BABY%', 'BABA', 'BT%', '%BT', '%(BT)%', '%[BT]%', '%B/%', '%TWIN%', '%TW1%', '%TW2%', '%TWN1%', '%TWN2%', '%T1%', '%T2%', '%TRIPLET%', '%QUADRUPLET%', '%GIRL/%', 'G/%', '%T/%', '%G/O%', '%BOY/%', '%/B%', '%B /O%', 'B TO%', '%BOY OF%', '%FETUS%', '%FOETUS%', '%BABAY%', '%BBAY %', '% BBAY%', '%BAB Y%', '%BY TO%', '%BEBY %', '%BABTY %', 'BBY %', '% BBY%', '% BAABY%', '%BAABY %', '%BABA TO%', '%BABA OF%', '%BANY %', 'BAY %', '%BABE TO%', '%BABE OF%', '%BT. %', '% BT.%', '%BARY TO%', '%BABHY %', '%BORN TO%', '%BORN OF%', '%BABUY TO%', '%BABUY OF%', '%BATY %', '%BABT %', '%BY BOY%', '%BY GIRL%', '%BAABY %', '%BABU OF%', '%GIRL %', '%CHILD OF%', '%CHILD TO%', '%UNKNOWN%', '%ABANDONE%'. Surname - '%BABY%', 'BT%', '%BT', '%(BT)%', '%[BT]%', '%B/%', '%TWIN %', '% TWIN%', '%TW1%', '%TW2%', '%TWN1%', '%TWN2%', '%T1%', '%T2%', '%TRIPLET%', '%QUADRUPLET%', '%GIRL/%', 'G/%', '%T/%', '%G/O%', '%BOY/%', '%/B%', '%B /O%', 'B TO%', '%BOY OF%', '%FETUS%', '%FOETUS%', '%BABAY %', '% BABAY%', '%BBAY %', '% BBAY%', '%BAB Y%', '%BY TO%', '%BEBY %', '%BABTY %', 'BBY %', ' BBY', '% BAABY%', '%BAABY %', '%BABA TO %', '%BABA OF %', '%BANY OF%', '%BANY TO%', '%BAY OF%', '%BAY TO%', '%BABE TO%', '%BABE OF%', '%BT. %', '% BT.%', '%BARY TO%', '%BABUY TO%', '%BABUY OF%', '%BATY %', '%BABT %', '%BY BOY%', '%BY GIRL%', '%BAABY %', '%BABU %', '%BABO %', '%GIRL %', '% GIRL%', '%CHILD OF%', '%CHILD TO%', '%ABANDONE%'. - *Given-name:* not *a Baby-derivative* name |

**Supplementary Table 1.** Derived Variables Used in Analysis.

*DDMMYY indicates the date, month and last two digits of the year of the patient’s DOB recorded in the system. ** ‘%’ indicates any number of additional characters.
